# Therapeutic Benefit in Allergic Dermatitis Derived from the Inhibitory Effect of Byakkokaninjinto on the Migration of Plasmacytoid Dendritic Cells

**DOI:** 10.1155/2020/9532475

**Published:** 2020-10-22

**Authors:** Takeshi Yamamoto, Yue Zhang, Ai Kigasawa, Shusaku Hayashi, Makoto Kadowaki

**Affiliations:** Division of Gastrointestinal Pathophysiology, Institute of Natural Medicine, University of Toyama, 2630 Sugitani, Toyama 930-0194, Japan

## Abstract

Dendritic cells (DCs) are well known to be essential immunocytes involved in innate and adaptive immunity. DCs are classified as conventional dendritic cells (cDCs) and plasmacytoid dendritic cells (pDCs). Recently, the accumulation of pDCs in inflamed tissues and lymphoid tissues has been considered to be a possible contributing factor in the development of immunological diseases, but little is known about the pathophysiological roles of pDCs in immunological diseases. To date, many studies have demonstrated that many kinds of Kampo formulas can regulate immunological reactions in human immune diseases. Thus, we screened Kampo formulas to identify an agent that inhibits pDC migration. Furthermore, we investigated the therapeutic effects of these formulas on a murine DNFB-induced allergic contact dermatitis model. Bone marrow-derived pDCs (BMpDCs) were derived from the bone marrow cells of BALB/c mice in a culture medium with Flt3 ligand. The effects of Kampo formulas on BMpDC migration were evaluated by assessing the number, velocity, and directionality of BMpDCs chemotaxing toward the more concentrated side of a chemokine (C-C motif) ligand 21 (CCL21) gradient. The Kampo formulas that exerted inhibitory effects on pDC migration were orally administered to DNFB-induced allergic contact dermatitis model mice. Byakkokaninjinto reduced the number of migrated BMpDCs and suppressed the velocity and directionality of BMpDC migration in a chemotaxis assay. Gypsum Fibrosum and Ginseng Radix, which are components of byakkokaninjinto, obviously suppressed the velocity of BMpDC migration. Furthermore, Gypsum Fibrosum significantly suppressed the directionality of BMpDC migration. In DNFB-induced allergic contact dermatitis model mice, byakkokaninjinto markedly abrogated ear swelling in late-phase allergic reactions. In conclusions, byakkokaninjinto, which has an inhibitory effect on pDC migration, was able to prevent the occurrence of allergic contact dermatitis, suggesting that pDCs were involved in the onset of allergic contact dermatitis in the mouse model. Therefore, byakkokaninjinto is anticipated to be a therapeutic agent for disorders related to pDC migration.

## 1. Introduction

Dendritic cells (DCs) play a critical role in immune regulation. In particular, DCs possess a strong ability to present antigens to CD4^+^ T cells. DCs, as versatile immunocytes, play roles in the induction of T-cell activation at the beginning of an immune response and in immune tolerance. During these processes, DCs need to migrate to organs and tissues to exert their functions. The migration of DCs is induced by chemokines and depends on chemokine concentration gradients [1]. In general, DCs have been divided into conventional dendritic cells (cDCs) and plasmacytoid dendritic cells (pDCs). The bone marrow is the primary site of pDC production, and pDCs migrate through the circulation to the thymus, secondary lymphoid organs, and peripheral tissues. In the normal state, pDCs rarely exist in peripheral tissues, but once viral infection occurs, pDCs accumulate in infected sites and rapidly secrete massive amounts of type 1 IFN. pDCs are activated by viruses; subsequently, they extend, form dendrites, and express the costimulators MHCII, CD40, CD80, and CD86 for antigen presentation [2]. Consequently, the migration of pDCs is important in facilitating the immune functions of these cells.

Chemokine (C-C motif) ligand 19 (CCL19) and chemokine (C-C motif) ligand 21 (CCL21), as ligands of C-C chemokine receptor type 7 (CCR7), are highly expressed in secondary lymphoid tissues [3]. The numbers of 120G8^+^ B220^int^ CD11c^int^ pDCs are decreased in the peripheral lymph nodes and mesenteric lymph nodes of CCR7-deficient mice [4]. Thus, CCR7 is required for pDC migration to the lymph nodes both under steady-state conditions and during viral infections [5].

pDCs have been reportedly related to inflammatory skin disorders [6]. Infiltrated pDCs have been found in the skin of systemic sclerosis patients, whereas pDCs in the peripheral blood have been found to be decreased, and depletion of B220^+^ PDCA-1^+^ pDCs reduces skin thickness in a skin fibrosis model [7]. Similarly, pDCs are increased, and pDCs accumulate in inflamed sites in patients with contact dermatitis [8, 9]. Otherwise, it has been reported that pDCs are increased in the lesional skin of atopic dermatitis patients [8–10]. Therefore, pDCs play essential roles in the pathogenic mechanism and defense mechanisms of many inflammatory skin disorders, including atopic dermatitis.

Kampo formulas have been used according to individual situations. Many Kampo formulas target the immune system, and their therapeutic effects on immunological diseases have been demonstrated in clinical studies [11, 12]. However, the precise mechanisms of Kampo formulas are not well understood. In addition, little is known about the effects of Kampo formulas on the function of DCs, a target for immunological diseases, even though DCs play a key role in immune responses.

Byakkokaninjinto, a traditional Japanese Kampo formula, has been frequently used in Japan and originates from the classical Chinese records of ‘Shang Han Lun' and ‘Jin Gui Yao Lue'. Byakkokaninjinto is composed of Gypsum Fibrosum, Ginseng Radix, Glycyrrhizae Radix, Anemarrhenae Rhizoma, and Oryzae Fructus (Table 1). Gypsum Fibrosum, as the basic component of byakkokaninjinto, is composed of calcium sulfate, and Kampo formulas containing Gypsum Fibrosum are used for their suppressive effects on heat and inflammation [13]. Anemarrhenae Rhizoma has anti-inflammatory effects [14]. Ginseng Radix has been used as an antiviral drug [15]. Therefore, byakkokaninjinto is widely applicable for the treatment of many disorders, such as oral dryness caused by diabetes, dermatitis, eczema, urticaria, pneumonia, and the common cold [16]. Dermatitis is one of the indications of byakkokaninjinto, and the efficacy of byakkokaninjinto in clinically curing atopic dermatitis and allergic contact dermatitis has been reported [16, 17]. In addition, it has been reported that byakkokaninjinto induces therapeutic effects on spontaneous atopic dermatitis-like skin lesions in NC mice [18] and IgE-mediated triphasic skin reaction [19]. However, the anti-inflammatory mechanism underlying the effects of byakkokaninjinto on these dermatitis remains uncertain.

In this study, we investigated the effects of 86 Kampo formulas on pDC migration and demonstrated that byakkokaninjinto exerted therapeutic effects on an DNFB-induced allergic contact dermatitis model by inhibiting pDC migration.

## 2. Materials and Methods

### 2.1. Mice

Male BALB/c mice (6–10 weeks old) were purchased from Japan SLC (Shizuoka, Japan). All mice were housed under standard vivarium conditions (23.5 ± 0.5°C, 12-hour light/dark cycle, and food and water provided ad libitum). This study was performed in strict accordance with the recommendations of the Guide for the Care and Use of Laboratory Animals by the National Institutes of Health. The Animal Experiment Committee at the University of Toyama approved all the animal care procedures and experiments (authorization numbers A2012 INM4, A2015 INM-3, and A2018 INM-4).

### 2.2. Preparation of Kampo Formulas and Each Component's Extracts

Kampo formula extracts and component extracts were provided as dried powders by the Joint Usage/Research Center for Science-Based Natural Medicine, Institute of Natural Medicine, University of Toyama, and the Knowledge Cluster Initiative Program (Second Stage) of the Ministry of Education, Culture, Sports, Science and Technology of Japan. Each herbal extract was obtained using a standard method. In brief, each formula and each herbal component was extracted in water at 100°C for 50 min, evaporated under reduced pressure, and freeze-dried to obtain a powder extract. The 86 Kampo formula extracts studied are shown in Table 2. Detailed information about the herbal components of the 86 Kampo formula extracts and detailed information of herbal component extracts are shown in Supplemental Tables 1 and 2.

For a mouse allergic contact dermatitis model study, Byakkokaninjinto (TJ-34) was purchased from Tsumura Co. (Tokyo, Japan) as a dried powder with the 3D-HPLC data (Supplemental Figure 1) and was evaluated as a therapeutic drug for the symptoms of DNFB-induced allergic contact dermatitis.

### 2.3. BMpDC Generation

Bone marrow-derived plasmacytoid dendritic cells (BMpDCs) were generated from bone marrow cells according to a method described previously [20]. Briefly, bone marrow cells were collected from the femur and tibia of male BALB/c mice (6–10 weeks old) and incubated in RPMI 1640 medium (Wako, Osaka, Japan) supplemented with 10% FBS (Equitech-Bio, Kerrville, TX, USA), 55 *μ*m 2-mercaptoethanol, 100 units/ml penicillin, 100 *μ*g/ml streptomycin, 292 *μ*g/ml glutamine (Invitrogen, Carlsbad, CA, USA), and 100 ng/ml Flt3 ligand (R&D Systems, Minneapolis, MN, USA). On days 7–9, immature BMpDCs were collected and stimulated with 2 *μ*m CpG-oligodeoxynucleotides (ODN-2216; Hokkaido System Science, Hokkaido, Japan) for 24 hours to induce maturation.

### 2.4. Chemotaxis Assay

Mature BMpDCs were suspended in modified RPMI 1640 medium (Sigma, St. Louis, MO, USA) containing 1% FBS and then incubated with each Kampo formula extract or herbal medicine extract for 3 hours at 37°C. Chemotaxis experiments with BMpDCs were performed in an EZ-TAXIScan^TM^ chamber according to the manufacturer's protocol (GE Healthcare Japan, Tokyo, Japan). A BMpDC suspension (1 × 10^6^ cells/ml) was injected into one side of the chamber, and 1 *μ*l of CCL21 (250 *μ*g/ml) was injected into the opposite side of the chamber. A concentration gradient of CCL21 was formed, and the migration of the BMpDCs toward the more concentrated side of a CCL21 gradient was observed. BMpDC migration was recorded with a CCD camera located beneath the chamber every 30 seconds for 1 hour. At the end of the chemotaxis assay, the number of migrated BMpDCs during 30 minutes and the velocity and directionality of the migrating BMpDCs were analyzed by the TAXIScan Analyzer 2.

### 2.5. Viability Assay and Analysis of CCR7 Expression Level

Treatment with byakkokaninjinto to mature BMpDCs was performed in the same way as the chemotaxis assay. Mature BMpDCs were suspended in modified RPMI 1640 medium containing 1% FBS and then incubated with byakkokaninjinto for 3 hours at 37°C. Subsequently, BMpDCs were stained with Via-Probe (Becton Dickinson, San Jose, CA, USA), anti-mouse mPDCA-1-APC (Miltenyi Biotec, Bergisch Gladbach, Germany), and anti-mouse CD11c-FITC (BD Biosciences, San Jose, CA, USA). To analyze the expression level of CCR7 on BMpDCs, BMpDCs were stained with anti-mouse CD197 (CCR7)-PE-Cy7 (eBioscience).

### 2.6. Flow Cytometry and Antibodies

Mature BMpDCs were suspended in FACS buffer (0.01 M phosphate-buffered saline (PBS) containing 1% BSA and 0.2% NaN_3_) and stained with the following antibodies: anti-mouse mPDCA-1-APC (Miltenyi Biotec), anti-mouse CD11c-FITC (BD Biosciences), anti-mouse MHC class II-PE (eBioscience, San Diego, CA, USA), and anti-mouse CD197 (CCR7)-PE-Cy7 (eBioscience). Cell proportions were analyzed with a BD FACSCantoII flow cytometer (BD Biosciences).

### 2.7. DNFB-Induced Allergic Contact Dermatitis Mouse Model

Male BALB/c mice (6 weeks old) were administered intraperitoneally 10 *μ*g DNP-OVA with 1 mg aluminum hydrogel on day 0. Byakkokaninjinto (0.5 g/kg or 1.0 g/kg) in 0.5% methylcellulose was administered orally on day 13. Skin lesions were induced by painting the ear with 0.1% 1-fluoro-2,4-dinitrobenzene (DNFB) in ethanol on day 14. Ear swelling induced with DNFB was evaluated by measuring ear thickness at 1 hour, 2 days, and 7 days after DNFB painting.

### 2.8. Statistical Analysis

The results are expressed as the mean ± SEM. Significant differences among groups were evaluated by one-way analysis of variance (ANOVA) followed by Dunnett's test for multiple comparisons, and significant differences between groups were evaluated with an unpaired Student's *t*-test. A *P* value less than 0.05 was considered significant.

## 3. Results

### 3.1. Effects of Kampo Formulas on the Migration of BMpDCs

**Figure 1:**
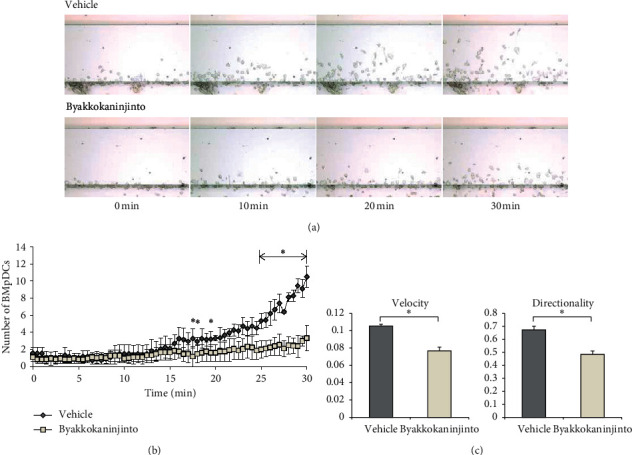
The effects of byakkokaninjinto on the migration of BMpDCs. Chemotactic responses were induced in BMpDCs by stimulation with the CCR7 ligand CCL21. Time-lapse images of the migration of BMpDCs treated with byakkokaninjinto (0.1 mg/ml) or the vehicle were recorded in an EZ-TAXIScan chemotaxis assay. (a) The number of byakkokaninjinto-treated BMpDCs that migrated during 30 minutes is indicated by the line graph and (b) data are expressed as the mean ± SE (^*∗*^*P* < 0.05, vs. the vehicle; *n* = 3). The effects of byakkokaninjinto on the velocity and directionality of BMpDC migration are represented by the bar chart; (c) data are expressed as the mean ± SE (^*∗*^*P* < 0.05, vs. the vehicle; *n* = 5).

To identify drugs that regulate DC migration, 86 Kampo formula extracts were screened using an EZ-TAXIScan chemotaxis assay. The chemotactic responses of BMpDCs were observed following stimulation with the CCR7 ligand CCL21, and BMpDCs migrated toward the more concentrated side of the CCL21 concentration gradient (Figure 1(a)). Among the 86 Kampo formula extracts, byakkokaninjinto significantly suppressed the migration of BMpDCs from 25 minutes to 30 minutes at each time point (Figures 1(a) and 1(b), *P* < 0.05), and shimotuto significantly accelerated the migration of BMpDCs (data not shown). The other 84 Kampo formula extracts did not show significant effects on BMpDC migration. Compared with the vehicle, byakkokaninjinto obviously decreased the number of migrated BMpDCs during 30 minutes (Figure 1(b)). In addition, we also performed a detailed analysis of the inhibitory effect of byakkokaninjinto by analyzing the velocity and directionality of BMpDC migration (Figure 1(c)). The velocity of the migration of BMpDCs treated with byakkokaninjinto (0.08 ± 0.00 *μ*m/sec) was significantly slower than that of BMpDCs treated with the vehicle (0.11 ± 0.00 *μ*m/sec; *P* < 0.05). In addition, the direction of BMpDC migration was calculated as the radian of cells migrating toward the more concentrated side of the concentration gradient of CCL21. The directionality of the migration of BMpDCs treated with byakkokaninjinto (0.48 ± 0.03 rad) was significantly lower than that of those treated with the vehicle (0.67 ± 0.03 rad; *P* < 0.05). These results indicate that byakkokaninjinto has a notable inhibitory effect on pDC migration.

### 3.2. Viability Assay with BMpDCs Treated with Byakkokaninjinto

**Figure 2:**
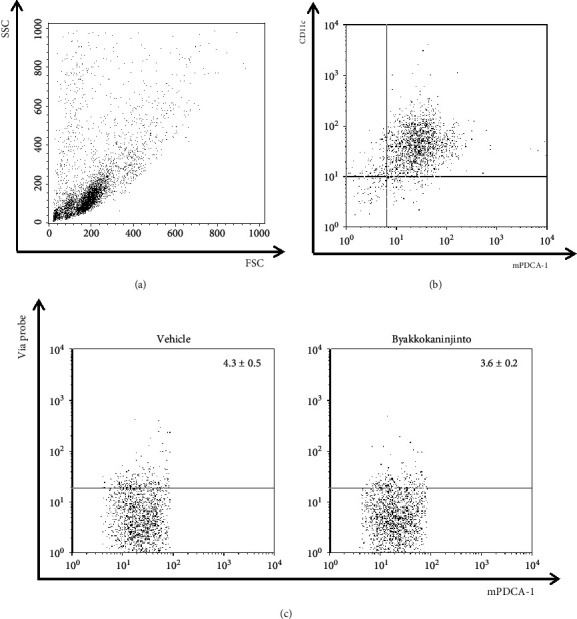
The detection of cell death caused by byakkokaninjinto. The toxicity of byakkokaninjinto to BMpDCs was detected by flow cytometry. The percentage of dead BMpDCs (CD11c^int^ mPDCA-1^+^ Via-Probe^+^) was determined. Dot plots show representative data. Data are expressed as the mean ± SE (*n* = 3).

To verify that the inhibitory effect of byakkokaninjinto on BMpDC migration is not due to toxic side effects, we examined the viability of BMpDCs treated with byakkokaninjinto. BMpDCs treated with byakkokaninjinto or the vehicle were stained with Via-Probe, an anti-CD11c antibody, and an anti-mPDCA-1 antibody. Dead BMpDCs (CD11c^int^ mPDCA-1^+^ Via-Probe^+^) were detected by flow cytometry. The proportion of dead BMpDCs following vehicle treatment was 4.3 ± 0.5%, and the proportion following byakkokaninjinto treatment was 3.6 ± 0.2% (Figure 2). Thus, byakkokaninjinto has no toxic side effects on BMpDCs.

### 3.3. Expression Level of CCR7 on BMpDCs Treated with Byakkokaninjinto

**Figure 3:**
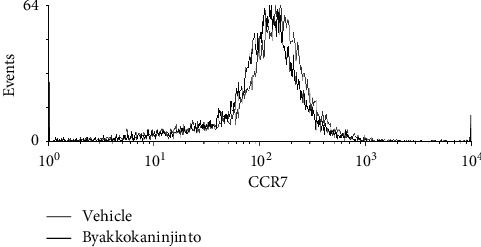
The effects of byakkokaninjinto on CCR7 expression in BMpDCs. The expression of CCR7 on BMpDCs treated with 0.1 mg/ml byakkokaninjinto (black line) or the vehicle (gray line) was detected by flow cytometry. Representative CCR7 expression data are represented by the line graph.

The maturation of BMpDCs is accompanied by the upregulation of CCR7 expression, which leads to increased sensitivity to CCL21. Thus, the expression level of CCR7 on BMpDCs was analyzed by flow cytometry. The expression level of CCR7 on BMpDCs treated with byakkokaninjinto was comparable to that on BMpDCs treated with the vehicle (Figure 3), indicating that byakkokaninjinto has no effect on the expression of CCR7 on BMpDCs.

### 3.4. Effects of the Components of Byakkokaninjinto on BMpDC Migration

**Figure 4:**
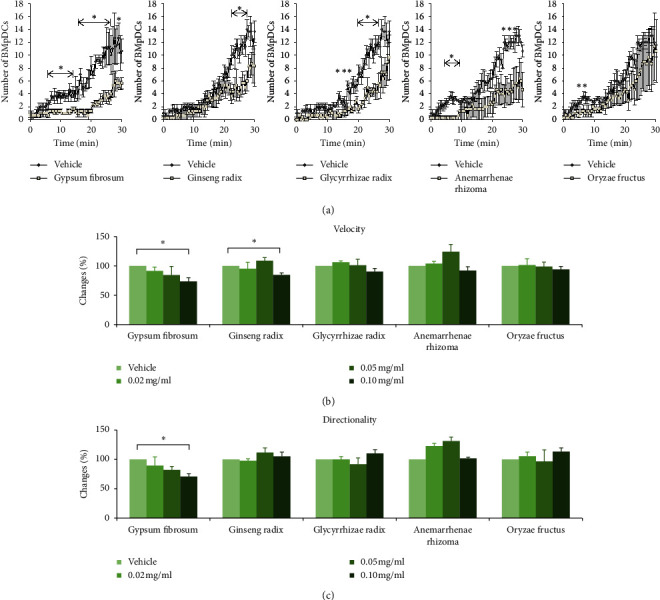
The effects of byakkokaninjinto components on BMpDC migration. The number of migrated BMpDCs with the treatment of each component (0.10 mg/ml) is indicated by the line graph; (a) data are expressed as the mean ± SE (^*∗*^*P* < 0.05, vs. the vehicle; *n* = 3). The effects of each component of byakkokaninjinto at doses of 0.02 mg/ml, 0.05 mg/ml, and 0.10 mg/ml on the velocity and directionality of BMpDC migration were measured by an EZ-TAXIScan chemotaxis assay. The bar chart shows the change rates of BMpDC migration velocity (b) and directionality (c) induced by treatment at each dose versus treatment with the vehicle. Data are expressed as the mean ± SE (^*∗*^*P* < 0.05, vs. the vehicle; *n* = 3).

Byakkokaninjinto is composed of Gypsum Fibrosum, Ginseng Radix, Glycyrrhizae Radix, Anemarrhenae Rhizoma, and Oryzae Fructus. We examined the effect of each component extract on the migration of BMpDCs. BMpDCs were treated with one component of byakkokaninjinto (0.02 mg/ml, 0.05 mg/ml, or 0.1 mg/ml) or the vehicle for 3 hours. The number of BMpDCs chemotaxing towards the CCL21 concentration gradient following treatment with each component extract or vehicle was increased during 30 minutes. At the dose of 0.1 mg/ml, the number of migrated BMpDCs was obviously decreased by the treatment of Gypsum Fibrosum, Ginseng Radix, and Glycyrrhizae Radix (Figure 4(a)) There was no effect of Anemarrhenae Rhizoma and Oryzae Fructus on the number of migrated BMpDCs (Figure 4(a)). To confirm the effect of each component, the value of the velocity and the directionality of BMpDC migration under treatment of each component of byakkokaninjinto (0.02 mg/ml, 0.05 mg/ml, or 0.1 mg/ml) were examined. The velocity of BMpDC migration in the vehicle treatment group was 0.12 ± 0.00 *μ*m/sec. Treatment with Gypsum Fibrosum (0.10 mg/ml) or Ginseng Radix (0.10 mg/ml) for 3 hours significantly reduced the velocity of BMpDC migration by 24.7% and 13.3%, respectively (Figure 4(b); *P* < 0.05). In addition, the inhibitory effect of Gypsum Fibrosum was dose-dependent.

The directionality of BMpDC migration in the vehicle group was 0.62 ± 0.04 rad. Treatment with Gypsum Fibrosum (0.10 mg/ml) significantly reduced the directionality of BMpDC migration by 37.5% (*P* < 0.05), and the inhibitory effect of Gypsum Fibrosum was dose-dependent (Figure 4(c)). Therefore, these results indicate that Gypsum Fibrosum is an active component in the inhibitory effect of byakkokaninjinto on BMpDC migration.

### 3.5. Effects of Byakkokaninjinto on an DNFB-Induced Allergic Contact Dermatitis Model

**Figure 5:**
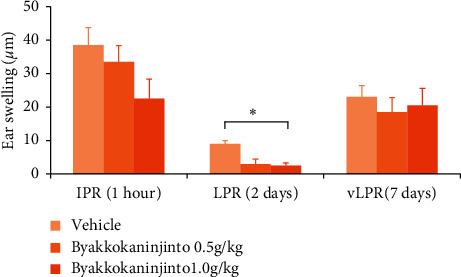
The effects of byakkokaninjinto on DNFB-induced allergic contact dermatitis model. Byakkokaninjinto (0.5 g/kg or 1.0 g/kg) was given by oral administration to DNFB-induced allergic contact dermatitis model mice 1 day before DNFB sensitization. Ear swelling was monitored during the IPR (1 hour), LPR (2 days), and vLPR (7 days). The bar chart shows the ear thicknesses of mice treated with 0.5 g/kg or 1.0 g/kg byakkokaninjinto or the vehicle during the IPR, LPR, and vLPR. Data are expressed as the mean ± SE (^*∗*^*P* < 0.05, vs. the vehicle; *n* = 5).

There are several inconsistent reports on the role of pDCs in the onset of dermatitis, which remains controversial. In the present study, we used a murine model of DNFB-induced allergic contact dermatitis that exhibits allergic contact dermatitis symptoms (ear swelling) with an immediate-phase reaction (IPR) at 1 hour, a late-phase reaction (LPR) at 2 days, and a very late-phase reaction (vLPR) at 6–10 days after DNFB administration [21]. Oral administration of byakkokaninjinto exhibited a tendency to suppress ear swelling during the IPR in a dose-dependent manner and then markedly prevented ear swelling during the LPR. However, byakkokaninjinto had no effect on ear swelling during the vLPR (Figure 5).

## 4. Discussion

The present study revealed that byakkokaninjinto inhibits the migration of BMpDCs and that Gypsum Fibrosum, one of the components of byakkokaninjinto, has a potent inhibitory effect on BMpDC migration. Furthermore, it suggested that pDCs are involved in the onset of allergic contact dermatitis and that byakkokaninjinto suppresses the onset of allergic contact dermatitis by inhibiting pDC migration.

Byakkokaninjinto significantly inhibited the migration of BMpDCs without inducing cellular toxicity in BMpDCs or enhancing the expression of CCR7 on BMpDCs. These results indicated that the inhibitory effect of byakkokaninjinto on BMpDC migration was caused by the inhibition of CCR7 signaling cascades. The activation of the CCR7 signaling pathway elicited by CCL21 is related to trimeric GTP-binding proteins, Jak family proteins, and Rho family proteins [3]. Rho, Rac, and Cdc42, members of the Rho family of proteins, play crucial roles in DC migration [22]. In particular, it has been reported that BMpDCs derived from DOCK2-deficient mice hardly migrate toward the more concentrated side of a concentration gradient of CCL21 [20]. Furthermore, Rac1 activation induced by CCL21 is almost abolished in DOCK2-deficient BMpDCs, although wild-type BMpDCs exhibit Rac1 activation [20]. Accordingly, Rho family proteins, including Rac1-DOCK2, are essential in the migration of pDCs. Thus, it is assumed that byakkokaninjinto inhibits the signaling pathway of Rac1-DOCK2 to suppress pDC migration.

We investigated the effect of each component of byakkokaninjinto on BMpDC migration and demonstrated that Gypsum Fibrosum and Ginseng Radix have inhibitory effects on BMpDC migration. However, the effective doses (0.1 mg/ml) of Gypsum Fibrosum and Ginseng Radix were higher than the amount contained in byakkokaninjinto. Moreover, makyokansekito, which contains the same dose of Gypsum Fibrosum as byakkokaninjinto, showed no inhibitory effect on the migration of pDCs. In addition, a high dose of Ginseng Radix is contained in many Kampo formulas, such as daikenchuto and shikunshito. However, these Kampo formulas had no effect on the migration of pDCs. In general, the pharmacological effects of Kampo formulas containing many herbal components are characterized by synergistic effects or additive effects of multiple herbal components and thus are complicated [23]. These results suggest that the inhibitory effect of byakkokaninjinto on pDC migration is attributed to the synergistic effects of multiple herbal medicines in byakkokaninjinto, including Gypsum Fibrosum and Ginseng Radix.

It has been reported that pDCs highly infiltrate the lesional skin of patients with contact dermatitis [8, 24] or atopic dermatitis [9, 10], whereas the proportion of CD304^+^ BDCA4^+^ pDCs does not change in the peripheral blood mononuclear cell population [9]. Conversely, BDCA2^+^ pDCs are not recruited into the lesional skin of patients with atopic dermatitis [24, 25]. Even in patients with atopic dermatitis, the proportion of CD123^+^ pDCs in the blood is higher than that in normal subjects [10]. In ovalbumin-induced dermatitis model mice, Wang et al. reported that the infiltration of pDCs was increased in the inflamed skin and that treatment with the immunostimulatory sequence CpG reduced pDC infiltration and skin inflammation [26], suggesting that pDCs play pivotal roles in the onset and development of dermatitis. However, in DNFB-induced dermatitis model similar to our used model, it is also reported that pDCs prevent the ear swelling response by inducing systemic tolerance [27, 28]. Accordingly, the role and distribution of pDCs in dermatitis remain poorly understood. In this study, we demonstrated that byakkokaninjinto had an inhibitory effect on pDC migration and suppressed the initiation of DNFB-induced allergic contact dermatitis. Therefore, the migration of pDCs to inflamed sites or the lymph nodes contributes to the onset and development of allergic contact dermatitis.

pDCs are well known for their capacity to present antigens [29–31] and secrete type 1 IFN in response to viral infections [2, 29]. Acquired immune responses are carried out through pDC migration to inflamed sites and mature pDC migration from the inflamed sites to the lymph nodes, where they perform antigen presentation. Consequently, the inhibition of pDC migration by byakkokaninjinto may have a beneficial effect on allergic dermatitis via the suppression of immune responses.

## 5. Conclusions

In conclusion, byakkokaninjinto possesses the ability to inhibit the migration of pDCs, thereby ameliorating allergic dermatitis. Moreover, byakkokaninjinto is anticipated to be used as a therapeutic agent for pDC-related diseases, such as allergic dermatitis.

## Figures and Tables

**Table 1:** Components and their percentages in byakkokaninjinto.

Components	Full-botanical plant names	Percentage
Gypsum Fibrosum	Natural hydrous calcium sulfate	48
Ginseng Radix	*Panax ginseng* C. A. Meyer	5
Glycyrrhizae Radix	*Glycyrrhiza uralensis* Fischer	6
Anemarrhenae Rhizoma	*Anemarrhena asphodeloides* Bunge	16
Oryzae Fructus	*Oryza sativa* Linne	25

**Table 2:** 86 kinds of Kampo prescriptions.

1	Anchusan
2	Inchinkoto
3	Eppikajutsuto
4	Orengedokuto
5	Kakkonto
6	Kamiuntanto
7	Kamikihito
8	Kamishoyosan
9	Kihito
10	Keishito
11	Keishibukuryogan
12	Goshuyuto
13	Goreisan
14	Saikokaryukotsuboreito
15	Saikokeishito
16	San'Oshashinto
17	Sansoninto
18	Shikunshito
19	Shimotsuto
20	Shakuyakukanzoto
21	Juzentaihoto
22	Shosaikoto
23	Shoseiryuto
24	Shimbuto
25	Daikenchuto
26	Daisaikoto
27	Chotosan
28	Tokakujokito
29	Tokishakuyakusan
30	Ninjinto
31	Bakumondoto
32	Hachimijiogan (decoction)
33	Hangekobokuto
34	Hangeshashito
35	Byakkokaninjinto
36	Boiogito
37	Bofutsushosan
38	Hochuekkito
39	Maoto
40	Maobushisaishinto
41	Unkeito
42	Unseiin
43	Ogikenchuto
44	Kambakutaisoto
45	Kyukikyogaito
46	Keigairengyoto
47	Keishikashakuyakuto
48	Keishikajutsubuto
49	Keishikaryukotsuboreito
50	Keishishakuyakuchimoto
51	Kososan
52	Goshajinkigan
53	Goshakusan
54	Saikokeishikankyoto
55	Saikoseikanto
56	Saibokuto
57	Saireito
58	Jiinkokato
59	Shigyakusan (decoction)
60	Shakanzoto
61	Jumihaidokuto
62	Shokenchuto
63	Shofusan
64	Seishinrenshiin
65	Seihaito
66	Sokeikakketsuto
67	Daiokanzoto
68	Daiobotampito
69	Daibofuto
70	Chikujountanto
71	Choijokito
72	Choreito
73	Tokishigyakukagoshuyushokyoto
74	Ninjin'Yoeito
75	Hangebyakujutsutemmato
76	Bukuryoin
77	Heiisan
78	Makyokansekito
79	Makyoyokukanto
80	Yokuininto
81	Yokukansan
82	Rikkunshito
83	Ryutanshakanto
84	Ryokyojutsukanto
85	Ryokeijutsukanto
86	Rokumigan (decoction)

## Data Availability

The data used to support the findings of this study are included within the article and are available from the corresponding author upon request.

## Supplementary Materials

Supplementary Materials Supplementary Figure 1: chemical profile of byakkokaninjinto analyzed by 3D-HPLC. Supplemental Table 1: components of 86 kinds of Kampo prescriptions. Supplemental Table 2: the details of herbal extracts in 86 kinds of Kampo prescriptions.
