# Supplementary material for: Therapeutic Benefit in Allergic Dermatitis Derived from the Inhibitory Effect of Byakkokaninjinto on the Migration of Plasmacytoid Dendritic Cells
Source: Evid Based Complement Alternat Med. 2020 Oct 22;2020:9532475. doi: 10.1155/2020/9532475 (PMC7603581; doi:10.1155/2020/9532475)
Supplement: Supplementary Materials — Supplementary Figure 1: chemical profile of byakkokaninjinto analyzed by 3D-HPLC. Supplemental Table 1: components of 86 kinds of Kampo prescriptions. Supplemental Table 2: the details of herbal extracts in 86 kinds of Kampo prescriptions. [file 9532475.f1.zip › Supplementary materials/Supplemental Figure 1.pdf]

Supplemental Figure

Supplemental Figure 1.

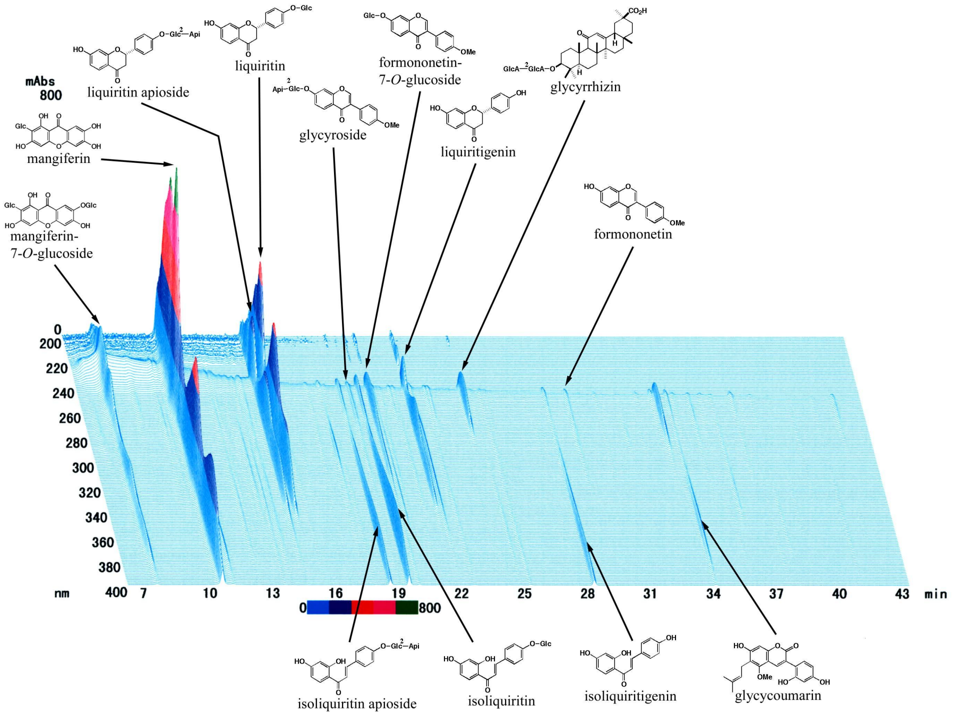

Supplemental Figure 1. Chemical profile of byakkokaninjinto analyzed by 3D-HPLC.

Each peak of byakkokaninjinto in the HPLC profile was identified by comparison of the retention times and UV spectra of chemically defined standard compounds. This 3D-HPLC data was created by Tsumura Co..
