# Supplementary material for: Therapeutic Benefit in Allergic Dermatitis Derived from the Inhibitory Effect of Byakkokaninjinto on the Migration of Plasmacytoid Dendritic Cells
Source: Evid Based Complement Alternat Med. 2020 Oct 22;2020:9532475. doi: 10.1155/2020/9532475 (PMC7603581; doi:10.1155/2020/9532475)
Supplement: Supplementary Materials — Supplementary Figure 1: chemical profile of byakkokaninjinto analyzed by 3D-HPLC. Supplemental Table 1: components of 86 kinds of Kampo prescriptions. Supplemental Table 2: the details of herbal extracts in 86 kinds of Kampo prescriptions. [file 9532475.f1.zip › Supplementary materials/Supplemental Table 1.docx]

| **Supplemental Table 1.** Components of 86 kinds of Kampo prescriptions | | |
| --- | --- | --- |
| **No** | **Kampo prescriptions** | **Components** |
| 1. | Anchusan | *Cinnamomi Cortex, Corydalis Tuber, Ostreae Testa, Foeniculi Fructus, Amomi Semen, Glycyrrhizae Radix, Alpiniae Officinari Rhizoma* |
| 2. | Inchinkoto | *Artemisiae Capillaris Flos, Gardeniae Fructus, Rhei Rhizoma* |
| 3. | Eppikajutsuto | *Ephedrae Herba, Gypsum Fibrosum, Zingiberis Rhizoma, Ziziphi Fructus, Glycyrrhizae Radix, Atractylodis Rhizoma* |
| 4. | Orengedokuto | *Ephedrae Herba, Gypsum Fibrosum, Zingiberis Rhizoma, Ziziphi Fructus, Glycyrrhizae Radix, Atractylodis Rhizoma* |
| 5. | Kakkonto | *Puerariae Radix, Ephedrae Herba, Zingiberis Rhizoma, Ziziphi Fructus, Cinnamomi Cortex, Paeoniae Radix, Glycyrrhizae Radix* |
| 6. | Kamiuntanto | *Pinelliae Tuber, Poria Sclerotium, Bambusae Caulis, Citri Unshiu Pericarpium, Ziziphi Semen, Glycyrrhizae Radix, Ziziphi Fructus, Aurantii Fructus Immaturus, Polygalae Radix, Scrophulariae Radix, Ginseng Radix, Rehmanniae Radix, Zingiberis Rhizoma* |
| 7. | Kamikihito | *Astragali Radix, Angelicae Acutilobae Radix, Gardeniae Fructus, Ginseng Radix, Atractylodis Rhizoma, Poria Sclerotium, Ziziphi Semen, Longan Arillus, Bupleuri Radix, Polygalae Radix, Ziziphi Fructus, Glycyrrhizae Radix, Zingiberis Rhizoma, Saussureae Radix* |
| 8. | Kamishoyosan | *Bupleuri Radix, Paeoniae Radix, Angelicae Acutilobae Radix, Poria Sclerotium, Gardeniae Fructus, Moutan Cortex, Glycyrrhizae Radix, Zingiberis Rhizoma, Menthae Herba, Atractylodis Rhizoma* |
| 9. | Kihito | *Astragali Radix, Angelicae Acutilobae Radix, Ginseng Radix, Atractylodis Rhizoma, Poria Sclerotium, Ziziphi Semen, Longan Arillus, Polygalae Radix, Ziziphi Fructus, Glycyrrhizae Radix, Zingiberis Rhizoma, Saussureae Radix* |
| 10. | Keishito | *Cinnamomi Cortex, Paeoniae Radix, Zingiberis Rhizoma, Ziziphi Fructus, Glycyrrhizae Radix* |
| 11. | Keishibukuryogan | *Cinnamomi Cortex, Poria Sclerotium, Paeoniae Radix, Persicae Semen, Moutan Cortex* |
| 12. | Goshuyuto | *Euodiae Fructus, Ziziphi Fructus, Zingiberis Rhizoma, Ginseng Radix* |
| 13. | Goreisan | *Alismatis Tuber, Poria Sclerotium, Polyporus, Atractylodis Rhizoma, Cinnamomi Cortex* |
| 14. | Saikokaryukotsuboreito | *Bupleuri Radix, Pinelliae Tuber, Poria Sclerotium, Cinnamomi Cortex, Ziziphi Fructus, Zingiberis Rhizoma, Ginseng Radix, Fossilia Ossis Mastodi, Ostreae Testa, Rhei Rhizoma* |
| 15. | Saikokeishito | *Bupleuri Radix, Pinelliae Tuber, Scutellariae Radix, Glycyrrhizae Radix, Cinnamomi Cortex, Paeoniae Radix, Ziziphi Fructus, Ginseng Radix, Zingiberis Rhizoma* |
| 16. | San’Oshashinto | *Rhei Rhizoma, Scutellariae Radix, Coptidis Rhizoma* |
| 17. | Sansoninto | *Ziziphi Semen, Poria Sclerotium, Anemarrhenae Rhizoma, Cnidii Rhizoma, Glycyrrhizae Radix* |
| 18. | Shikunshito | *Ginseng Radix, Atractylodis Rhizoma, Poria Sclerotium, Glycyrrhizae Radix, Ziziphi Fructus, Zingiberis Rhizoma* |
| 19. | Shimotsuto | *Angelicae Acutilobae Radix, Paeoniae Radix, Cnidii Rhizoma, Rehmanniae Radix* |
| 20. | Shakuyakukanzoto | *Paeoniae Radix, Glycyrrhizae Radix* |
| 21. | Juzentaihoto | *Astragali Radix, Cinnamomi Cortex, Paeoniae Radix, Angelicae Acutilobae Radix, Rehmanniae Radix, Cnidii Rhizoma, Ginseng Radix, Poria Sclerotium, Atractylodis Rhizoma, Glycyrrhizae Radix* |
| 22. | Shosaikoto | *Bupleuri Radix, Pinelliae Tuber, Scutellariae Radix, Ginseng Radix, Ziziphi Fructus, Glycyrrhizae Radix, Zingiberis Rhizoma* |
| 23. | Shoseiryuto | *Ephedrae Herba, Cinnamomi Cortex, Paeoniae Radix, Asiasari Radix, Zingiberis Processum Rhizoma, Schisandrae Fructus, Pinelliae Tuber, Glycyrrhizae Radix* |
| 24. | Shimbuto | *Poria Sclerotium, Paeoniae Radix, Atractylodis Rhizoma, Zingiberis Rhizoma, Aconiti Radix* |
| 25. | Daikenchuto | *Ginseng Radix, Zingiberis Processum Rhizoma, Zanthoxyli Piperiti Pericarpium, Koi* |
| 26. | Daisaikoto | *Bupleuri Radix, Pinelliae Tuber, Scutellariae Radix, Paeoniae Radix, Ziziphi Fructus, Aurantii Fructus Immaturus, Zingiberis Rhizoma, Rhei Rhizoma* |
| 27. | Chotosan | *Gypsum Fibrosum, Uncariae Uncis cum Ramulus, Citri Unshiu Pericarpium, Pinelliae Tuber, Ophiopogonis Radix, Ophiopogonis Radix, Ophiopogonis Radix, Chrysanthemi Flos, Saposhnikoviae Radix, Glycyrrhizae Radix, Zingiberis Rhizoma* |
| 28. | Tokakujokito | *Persicae Semen, Rhei Rhizoma, Cinnamomi Cortex, Glycyrrhizae Radix, Sal Mirabilis* |
| 29. | Tokishakuyakusan | *Paeoniae Radix, Atractylodis Rhizoma, Alismatis Tuber, Poria Sclerotium, Cnidii Rhizoma, Angelicae Acutilobae Radix* |
| 30. | Ninjinto | *Ginseng Radix, Glycyrrhizae Radix, Zingiberis Processum Rhizoma, Atractylodis Rhizoma* |
| 31. | Bakumondoto | *Ophiopogonis Radix, Pinelliae Tuber, Oryzae Fructus, Ziziphi Fructus, Ginseng Radix, Glycyrrhizae Radix* |
| 32. | Hachimijiogan (decoction) | *Rehmanniae Radix, Corni Fructus, Dioscoreae Rhizoma, Alismatis Tuber, Poria Sclerotium, Moutan Cortex, Cinnamomi Cortex, Aconiti Radix* |
| 33. | Hangekobokuto | *Pinelliae Tuber, Poria Sclerotium, Magnoliae Cortex, Perilla Herba, Zingiberis Rhizoma* |
| 34. | Hangeshashito | *Pinelliae Tuber, Scutellariae Radix, Glycyrrhizae Radix, Zingiberis Processum Rhizoma, Ginseng Radix, Ziziphi Fructus, Coptidis Rhizoma* |
| 35. | Byakkokaninjinto | *Gypsum Fibrosum, Anemarrhenae Rhizoma, Oryzae Fructus, Glycyrrhizae Radix, Ginseng Radix* |
| 36. | Boiogito | *Sinomeni Caulis et Rhizoma, Astragali Radix, Atractylodis Rhizoma, Ziziphi Fructus, Zingiberis Rhizoma, Glycyrrhizae Radix* |
| 37. | Bofutsushosan | *Rhei Rhizoma, Sal Mirabilis, Ephedrae Herba, Saposhnikoviae Radix, Schizonepetae Spica, Menthae Herba, Kasseki, Gardeniae Fructus, Gypsum Fibrosum, Platycodi Radix, Forsythiae Fructus, Scutellariae Radix, Cnidii Rhizoma, Angelicae Acutilobae Radix, Paeoniae Radix, Atractylodis Rhizoma, Glycyrrhizae Radix, Zingiberis Rhizoma* |
| 38. | Hochuekkito | *Ginseng Radix, Atractylodis Rhizoma, Astragali Radix, Angelicae Acutilobae Radix, Bupleuri Radix, Citri Unshiu Pericarpium, Ziziphi Fructus, Zingiberis Rhizoma, Glycyrrhizae Radix, Cimicifugae Rhizoma* |
| 39. | Maoto | *Ephedrae Herba, Armeniacae Semen, Cinnamomi Cortex, Glycyrrhizae Radix* |
| 40. | Maobushisaishinto | *Aconiti Radix, Asiasari Radix, Ephedrae Herba* |
| 41. | Unkeito | *Pinelliae Tuber, Ophiopogonis Radix, Angelicae Acutilobae Radix, Paeoniae Radix, Cnidii Rhizoma, Asini Corii Collas, Moutan Cortex, Ginseng Radix, Cinnamomi Cortex, Glycyrrhizae Radix, Zingiberis Rhizoma, Euodiae Fructus* |
| 42. | Unseiin | *Angelicae Acutilobae Radix, Processi Rehmanniae Radix, Paeoniae Radix, Cnidii Rhizoma, Scutellariae Radix, Gardeniae Fructus, Coptidis Rhizoma, Phellodendri Cortex* |
| 43. | Ogikenchuto | *Cinnamomi Cortex, Zingiberis Rhizoma, Ziziphi Fructus, Astragali Radix, Paeoniae Radix, Glycyrrhizae Radix, Koi* |
| 44. | Kambakutaisoto | *Glycyrrhizae Radix, Ziziphi Fructus, Tritici Semen* |
| 45. | Kyukikyogaito | *Rehmanniae Radix, Angelicae Acutilobae Radix, Paeoniae Radix, Cnidii Rhizoma, Asini Corii Collas, Artemisiae Folium, Glycyrrhizae Radix* |
| 46. | Keigairengyoto | *Angelicae Acutilobae Radix, Paeoniae Radix, Cnidii Rhizoma, Rehmanniae Radix, Coptidis Rhizoma, Phellodendri Cortex,Scutellariae Radix, Gardeniae Fructus, Forsythiae Fructus, Saposhnikoviae Radix, Menthae Herba, Schizonepetae Spica, Glycyrrhizae Radix, Aurantii Pericarpium, Bupleuri Radix, Angelicae Dahuricae Radix, Platycodi Radix* |
| 47. | Keishikashakuyakuto | *Cinnamomi Cortex, Paeoniae Radix, Zingiberis Rhizoma, Ziziphi Fructus, Glycyrrhizae Radix* |
| 48. | Keishikajutsubuto | *Cinnamomi Cortex, Paeoniae Radix, Zingiberis Rhizoma, Ziziphi Fructus, Glycyrrhizae Radix, Atractylodis Rhizoma, Aconiti Radix* |
| 49. | Keishikaryukotsuboreito | *Cinnamomi Cortex, Paeoniae Radix, Zingiberis Rhizoma, Ziziphi Fructus, Glycyrrhizae Radix, Fossilia Ossis Mastodi, Ostreae Testa* |
| 50. | Keishishakuyakuchimoto | *Ephedrae Herba, Saposhnikoviae Radix, Cinnamomi Cortex, Paeoniae Radix, Zingiberis Rhizoma, Anemarrhenae Rhizoma, Atractylodis Rhizoma, Glycyrrhizae Radix, Aconiti Radix* |
| 51. | Kososan | *Cyperi Rhizoma, Perilla Herba, Citri Unshiu Pericarpium, Glycyrrhizae Radix, Zingiberis Rhizoma* |
| 52. | Goshajinkigan | *Rehmanniae Radix, Achyranthis Radix, Cornus Fruit, Dioscoreae Rhizoma, Plantaginis Semen, Alismatis Tuber, Poria Sclerotium, Moutan Cortex, Aconiti Radix, Cinnamomi Cortex* |
| 53. | Goshakusan | *Atractylodis Rhizoma, Poria Sclerotium, Tachibana Pericarpium, Pinelliae Tuber, Magnoliae Cortex, Aurantii Fructus Immaturus, Platycodi Radix, Angelicae Dahuricae Radix, Glycyrrhizae Radix, Ephedrae Herba, Cinnamomi Cortex, Zingiberis Processum Rhizoma, Angelicae Acutilobae Radix, Paeoniae Radix, Cnidii Rhizoma* |
| 54. | Saikokeishikankyoto | *Bupleuri Radix, Cinnamomi Cortex, Scutellariae Radix, Ostreae Testa, Zingiberis Processum Rhizoma, Glycyrrhizae Radix, Trichosanthis Radix* |
| 55. | Saikoseikanto | *Bupleuri Radix, Angelicae Acutilobae Radix, Paeoniae Radix, Cnidii Rhizoma, Rehmanniae Radix, Coptidis Rhizoma, Scutellariae Radix, Phellodendri Cortex, Gardeniae Fructus, Forsythiae Fructus, Platycodi Radix, Arctii Fructus, Trichosanthis Radix, Menthae Herba, Glycyrrhizae Radix* |
| 56. | Saibokuto | *Bupleuri Radix, Pinelliae Tuber, Zingiberis Rhizoma, Scutellariae Radix, Ziziphi Fructus, Ginseng Radix, Glycyrrhizae Radix, Magnoliae Cortex, Poria Sclerotium, Perilla Herba* |
| 57. | Saireito | *Bupleuri Radix, Alismatis Tuber, Pinelliae Tuber, Scutellariae Radix, Atractylodis Rhizoma, Ziziphi Fructus, Polyporus, Ginseng Radix, Poria Sclerotium, Glycyrrhizae Radix, Cinnamomi Cortex, Zingiberis Rhizoma* |
| 58. | Jiinkokato | *Atractylodis Rhizoma, Rehmanniae Radix, Paeoniae Radix, Citri Unshiu Pericarpium, Angelicae Acutilobae Radix, Ophiopogonis Radix, Phellodendri Cortex, Glycyrrhizae Radix, Anemarrhenae Rhizoma, Asparagi Radix* |
| 59. | Shigyakusan (decoction) | *Bupleuri Radix, Paeoniae Radix, Aurantii Fructus Immaturus, Glycyrrhizae Radix* |
| 60. | Shakanzoto | *Glycyrrhizae Radix Praeparata, Zingiberis Rhizoma, Cinnamomi Cortex, Cannabidis Fructus, Ziziphi Fructus, Ginseng Radix, Rehmanniae Radix, Ophiopogonis Radix, Asini Corii Collas* |
| 61. | Jumihaidokuto | *Bupleuri Radix, Platycodi Radix, Pruni Cortex, Saposhnikoviae Radix, Poria Sclerotium, Cnidii Rhizoma, Araliae Cordatae Rhizoma, Schizonepetae Spica, Glycyrrhizae Radix, Zingiberis Rhizoma* |
| 62. | Shokenchuto | *Paeoniae Radix, Cinnamomi Cortex, Ziziphi Fructus, Glycyrrhizae Radix, Zingiberis Rhizoma, Koi* |
| 63. | Shofusan | *Angelicae Acutilobae Radix, Rehmanniae Radix, Gypsum Fibrosum, Saposhnikoviae Radix, Atractylodis Lanceae Rhizoma, Akebiae Caulis, Arctii Fructus, Anemarrhenae Rhizoma, Sesami Semen, Glycyrrhizae Radix, Cicadae Periostracum, Sophorae Radix, Schizonepetae Spica* |
| 64. | Seishinrenshiin | *Ophiopogonis Radix, Poria Sclerotium, Nelumbis Semen, Ginseng Radix, Plantaginis Semen, Scutellariae Radix, Astragali Radix, Lycii Cortex, Glycyrrhizae Radix* |
| 65. | Seihaito | *Angelicae Acutilobae Radix, Ophiopogonis Radix, Poria Sclerotium, Scutellariae Radix, Platycodi Radix, Armeniacae Semen, Gardeniae Fructus, Mori Cortex, Ziziphi Fructus, Citri Unshiu Pericarpium, Bambusae Caulis, Asparagi Radix, Fritillariae Bulbus, Glycyrrhizae Radix, Schisandrae Fructus, Zingiberis Rhizoma* |
| 66. | Sokeikakketsuto | *Paeoniae Radix, Rehmanniae Radix, Cnidii Rhizoma, Atractylodis Lanceae Rhizoma, Angelicae Acutilobae Radix, Persicae Semen, Poria Sclerotium, Achyranthis Radix, Citri Unshiu Pericarpium, Sinomeni Caulis et Rhizoma, Saposhnikoviae Radix, Gentianae Scabrae Radix, Clematidis Radix, Notopterygii Rhizoma, Glycyrrhizae Radix, Angelicae Dahuricae Radix, Zingiberis Rhizoma* |
| 67. | Daiokanzoto | *Rhei Rhizoma, Glycyrrhizae Radix* |
| 68. | Daiobotampito | *Benincasae Semen, Rhei Rhizoma, Persicae Semen, Moutan Cortex, Sal Mirabilis* |
| 69. | Daibofuto | *Angelicae Acutilobae Radix, Paeoniae Radix, Rehmanniae Radix, Astragali Radix, Saposhnikoviae Radix, Eucommiae Cortex, Atractylodis Rhizoma, Cnidii Rhizoma, Ginseng Radix, Notopterygii Rhizoma, Achyranthis Radix, Glycyrrhizae Radix, Ziziphi Fructus, Zingiberis Rhizoma, Aconiti Radix* |
| 70. | Chikujountanto | *Pinelliae Tuber, Citri Unshiu Pericarpium, Poria Sclerotium, Glycyrrhizae Radix, Aurantii Fructus Immaturus, Bambusae Caulis, Ziziphi Fructus, Bupleuri Radix, Coptidis Rhizoma, Cyperi Rhizoma, Platycodi Radix, Ophiopogonis Radix, Ginseng Radix* |
| 71. | Choijokito | *Rhei Rhizoma, Sal Mirabilis, Glycyrrhizae Radix* |
| 72. | Choreito | *Polyporus, Poria Sclerotium, Kasseki, Alismatis Tuber, Asini Corii Collas* |
| 73. | Tokishigyakukagoshuyushokyoto | *Ziziphi Fructus, Angelicae Acutilobae Radix, Paeoniae Radix, Cinnamomi Cortex, Akebiae Caulis, Glycyrrhizae Radix, Asiasari Radix, Euodiae Fructus, Zingiberis Rhizoma* |
| 74. | Ninjin’Yoeito | *Ginseng Radix, Astragali Radix, Atractylodis Rhizoma, Poria Sclerotium, Angelicae Acutilobae Radix, Rehmanniae Radix, Cinnamomi Cortex, Paeoniae Radix, Citri Unshiu Pericarpium, Polygalae Radix, Schisandrae Fructus, Glycyrrhizae Radix* |
| 75. | Hangebyakujutsutemmato | *Pinelliae Tuber, Citri Unshiu Pericarpium, Fructus Hordei Germinatus, Poria Sclerotium, Astragali Radix, Ginseng Radix, Alismatis Tuber, Atractylodis Rhizoma, Atractylodis Lanceae Rhizoma, Gastrodiae Tuber, Massa Medicata Fermentata, Phellodendri Cortex, Zingiberis Rhizoma, Zingiberis Processum Rhizoma* |
| 76. | Bukuryoin | *Poria Sclerotium, Atractylodis Rhizoma, Ginseng Radix, Zingiberis Rhizoma, Tachibana Pericarpium, Aurantii Fructus Immaturus* |
| 77. | Heiisan | *Atractylodis Lanceae Rhizoma, Magnoliae Cortex, Citri Unshiu Pericarpium, Ziziphi Fructus, Zingiberis Processum Rhizoma, Glycyrrhizae Radix* |
| 78. | Makyokansekito | *Ephedrae Herba, Armeniacae Semen, Glycyrrhizae Radix, Gypsum Fibrosum* |
| 79. | Makyoyokukanto | *Ephedrae Herba, Armeniacae Semen, Coicis Semen, Glycyrrhizae Radix* |
| 80. | Yokuininto | *Ephedrae Herba, Angelicae Acutilobae Radix, Atractylodis Rhizoma, Coicis Semen, Cinnamomi Cortex, Paeoniae Radix, Glycyrrhizae Radix* |
| 81. | Yokukansan | *Atractylodis Rhizoma, Poria Sclerotium, Angelicae Acutilobae Radix, Cnidii Rhizoma, Uncariae Uncis cum Ramulus, Bupleuri Radix, Glycyrrhizae Radix* |
| 82. | Rikkunshito | *Ginseng Radix, Atractylodis Rhizoma, Poria Sclerotium, Glycyrrhizae Radix, Citri Unshiu Pericarpium, Pinelliae Tuber, Ziziphi Fructus, Zingiberis Rhizoma* |
| 83. | Ryutanshakanto | *Gentianae Scabrae Radix, Angelicae Acutilobae Radix, Rehmanniae Radix, Alismatis Tuber, Akebiae Caulis, Plantaginis Semen, Scutellariae Radix, Gardeniae Fructus, Glycyrrhizae Radix* |
| 84. | Ryokyojutsukanto | *Poria Sclerotium, Atractylodis Rhizoma, Zingiberis Processum Rhizoma, Glycyrrhizae Radix* |
| 85. | Ryokeijutsukanto | *Poria Sclerotium, Cinnamomi Cortex, Atractylodis Rhizoma, Glycyrrhizae Radix* |
| 86. | Rokumigan (decoction) | *Rehmanniae Radix, Corni Fructus, Dioscoreae Rhizoma, Alismatis Tuber, Poria Sclerotium, Moutan Cortex, Cinnamomi Cortex, Aconiti Radix, Alismatis Tuber, Poria Sclerotium, Moutan Cortex* |
